# Looking over the rim: algorithms for cheminformatics from computer scientists

**DOI:** 10.1186/1758-2946-6-S1-O13

**Published:** 2014-03-11

**Authors:** Thorsten Meinl, Bernd Wiswedel, Michael R Berthold

**Affiliations:** 1 KNIME.com AG, Zurich, 8005, Switzerland; 2 University of Konstanz, Konstanz, 78457, Germany

## 

In recent years a number of methods were invented in the data mining/machine learning field that have received little attention in the cheminformatics world even though they offer interesting properties for these types of applications as well - even compared to some similar algorithms published primarily in the cheminformatics space. In this talk we want to highlight three of these algorithms/approaches. The first is MoSS [1], a frequent subgraph miner that can not only be used to find common substructures in a set of molecules but is also able to compute the MCSS very fast and has some extension especially suited for molecules. The second presented approach deals with the problem of finding diverse subsets of molecules [2]. Quite interestingly, not only finding a diverse subset can be a challenging task but already the definition of diversity is not as straight-forward as it seems at the first glance. The third algorithm goes along the same lines but tries to find similar molecules by looking at their properties from so-called *parallel universes*[3]. Each universe contains a set of related properties and partial predictive models are built in each universe separately. Through interactive model construction, e.g. by so-called Neighbourgrams, the models from one universe can aid the construction of a models in other universes.
